# Prediction of Compressive Strength of Concrete Specimens Based on Interpretable Machine Learning

**DOI:** 10.3390/ma17153661

**Published:** 2024-07-24

**Authors:** Wenhu Wang, Yihui Zhong, Gang Liao, Qing Ding, Tuan Zhang, Xiangyang Li

**Affiliations:** Power China Chengdu Engineering Corporation Ltd., Chengdu 610031, China; p2024806@chidi.com.cn (W.W.); 2005051@chidi.com.cn (Y.Z.); 2012037@chidi.com.cn (Q.D.); p2024804@chidi.com.cn (X.L.)

**Keywords:** compressive strength, machine learning, XGBoost, SHAP

## Abstract

The aim of this paper is to explore an effective model for predicting the compressive strength of concrete using machine learning technology, as well as to interpret the model using an interpretable method, which overcomes the limitation of the unknowable prediction processes of previous machine learning models. An experimental database containing 228 samples of the compressive strength of standard cubic specimens was built in this study, and six algorithms were applied to build the predictive model. The results show that the XGBoost model has the highest prediction accuracy among all models, as the R^2^ of the training set and testing set are 0.982 and 0.966, respectively. Further analysis was conducted on the XGBoost model to discuss its applicability. The main steps include the following: (i) obtaining key features, (ii) obtaining trends in the evolution of features, (iii) single-sample analysis, and (iv) conducting a correlation analysis to explore methods of visualizing the variations in the factors that exert influence. The interpretability analyses on the XGBoost model show that the contribution to the compressive strength by each factor is highly in line with the conventional theory. In summary, the XGBoost model proved to be effective in predicting concrete’s compressive strength.

## 1. Introduction

Concrete is a ubiquitous material in civil engineering, and determining its compressive strength accurately is crucial to engineering construction and operation. As a composite material, it is challenging to accurately predict its compressive strength due to its complex and variable composition, differences in curing times, and curing environments. Currently, the main methods for obtaining the compressive strength of concrete are the rebound method, core drilling method, and model prediction method. The rebound method is easy to carry out; however, using different rebound meters for the same type of concrete can yield large discrepancies and fail to accurately estimate the internal strength. The core drilling method is time-consuming, and the results are not stable. Predictive modeling involves creating mathematical models that correlate the material mix, age, and measured compressive strength, but due to the nonlinear relationships between these factors, the establishment of an explicit mathematical model is more difficult. Therefore, there is an urgent need for a fast, accurate, and reliable method for predicting the compressive strength of concrete.

With advances in artificial intelligence and big data analytics, some of these challenges are being mitigated. Machine learning algorithms, including decision trees (DTs), Naive Bayes, and ensemble methods such as Random Forest, AdaBoost, Gradient Boosting Decision Trees (GBDTs), and XGBoost, have been increasingly employed. For instance, Rankbar et al. [1] utilized a deep learning algorithm and evaluated six models with varying hyperparameters. Adhikary et al. [2] developed an artificial neural network model using five input features that provided a more accurate prediction of the ultimate shear strength in reinforced concrete than in traditional formulas. Mansouri et al. [3,4] applied two algorithms, ANN and ANFIS, successfully predicting the debonding strength of masonry. Extensive experimentation by previous researchers on concrete’s compressive strength illustrates the potential of machine learning algorithms to significantly enhance data utility. Nevertheless, current research has not adequately addressed how variations in input parameters influence prediction outcomes. This gap highlights the growing need for methods that can elucidate the predictive modeling process, balancing accuracy with interpretability. Therefore, constructing a precise mathematical model is challenging.

In order to improve the credibility of machine learning models, a variety of interpretive methods have been proposed. Among these methods, the SHAP method is a more comprehensive interpretable method. It includes global and local interpretations. Local interpretation, or single-sample analysis, quantifies the contribution of each input variable to the predicted values, whereas global interpretation assesses feature importance and dependency. For example, Feng et al. [5] aligned the feature importance rankings in a deep-beam prediction model with established theories. Mangalathu S et al. [6] performed an interpretive analysis for seismic performance assessment, and further work by Mangalathu S et al. [7,8,9,10,11,12,13,14,15,16,17,18,19,20,21,22,23] clarified the importance of specific characteristics and their impact on the seismic performance of infrastructures using SHAP. Bentz predicted changes in the heat release, chemical shrinkage, and compressive strength over time using kinetic calibration [24,25,26,27,28]. The method suggested by Mechling et al. greatly improves the accuracy of slurry strength calculations. These results can be used to calculate concrete’s strength [29,30,31,32]. Abuodeh et al. used ANN with Sequence Feature Selection (SFS) and a Neural Interpretation Diagram (NID) to greatly improve the accuracy of the model and provide valuable insights into the ANN’s compressive strength predictions of different UHPC mixes [33,34,35,36]. However, the ambiguous influence patterns of input features on concrete’s compressive strength have so far limited the full validation of interpretive analyses in machine learning models.

Accordingly, this study employed various machine learning algorithms to predict concrete’s compressive strength, verifying the soundness of the prediction model through the analysis of key features, trends in feature evolution, single-sample interpretation, and a correlation analysis.

## 2. Research Methods and Modeling Process

### 2.1. Algorithmic Principle

In this study, six algorithms were selected to predict the concrete’s compressive strength. By comparing the six algorithms, the best performing prediction model was obtained. The principles of the selected algorithms are briefly described below.

K Nearest Neighbors (KNN) is one of the simplest methods in data mining techniques; to predict the target value based on the distance between data points, it selects K nearest neighbor data points and then uses their labels or averages to classify or regress them [37,38]. A Decision Tree (DT) is a supervised learning method that can summarize the decision rules from a series of featured and labeled data and present these rules in a tree-like structure to solve classification and regression problems. The main parameters of the model are the maximum depth of the tree, maximum number of leaf nodes, minimum number of partitioned samples, and so on [39].

Random Forest (RF) [40,41] is an integrated algorithm based on bagging, which combines multiple decision trees, where the dataset is selected randomly and put back each time, and some of the features are also randomly selected as inputs. RF reduces the risk of overfitting a single tree by integrating multiple decision trees. Each tree sees only part of the data and some of the features, thus improving the generalization of the overall model.

A boosting algorithm boosts a weak learner to a strong learner algorithm by means of concatenation [42]. Depending on the strategy, there are three common integrated algorithms, namely GBDT [43], Adaboost [44], and XGBoost [45,46]. GBDT is designed to continuously reduce the residuals (regression) by continuously adding new trees designed to build a new model in the direction of residual reduction (negative gradient). The loss function is designed to reduce the residuals as fast as possible. In order to obtain the residuals, all decision trees use CART regression trees. Adaboost emphasizes adaptability by constantly modifying the sample weights (increasing the weights of the wrong samples and decreasing the weights of the right samples) and constantly adding weak classifiers for boosting. The principle of XGBoost is similar to that of GBDT, which is optimized as a distributed gradient. XGBoost is an optimized distributed gradient boosting library and is also a tool for massively parallel boosting trees. Compared with GBDT [47], XGBoost has three main improvements: (1) XGBoost takes into account the complexity of the tree, while GBDT does not; (2) XGBoost fits the second-order derivative expansion of the loss function of the previous round, while GBDT fits the first-order derivative of the loss function of the previous round, and thus, the former has fewer iterations and is more accurate; and (3) XGBoost has a faster running speed when selecting the optimal cut-points as it uses multi-threading to carry out the process.

In summary, the first two are single algorithms, and the last four are integrated algorithms. While RF is a bagging-class integrated algorithm, GBDT, Adaboost, and XGBoost are boosting-class integrated algorithms, and the best performing model is selected by comparing the six algorithms.

### 2.2. Evaluation Metrics

For regression tasks, the common evaluation metrics are the MSE (Mean Square Error), MAE (Mean Absolute Error), RMSE (Root Mean Square Error), MAPE (Mean Absolute Percentage Error), and the Coefficient of determination R-Squared (R^2^). And both the MSE and MAE are measures of the gap between the predicted value and the true value. Therefore, the latter four were chosen in this paper as the indicators for evaluating the model. Here, four commonly used evaluation metrics were selected and defined as follows:

--R^2^:

(1) $$R^{2}=1-\frac{{\sum_{i=1}^{m}(P_{i}-T_{i})}^{2}}{{\sum_{i=1}^{m}(P_{i}-\bar{T})}^{2}}$$

--RMSE:

(2) $$\mathrm{RMSE}=\sqrt{\frac{{\sum_{i=1}^{m}(P_{i}-T_{i})}^{2}}{m}}$$

--MAE:

(3) $$\mathrm{MAE}=\frac{\sum_{i=1}^{m}\left|P_{i}-T_{i}\right|}{m}$$

--MAPE:

(4) $$\mathrm{MAPE}=\frac{100\%}{m}\sum_{i=1}^{m}\left|P_{i}-T_{i}\right|$$

### 2.3. The Interpretable Method

Lundberg and Lee [48,49,50] proposed the interpretable method known as the Shapley additive explanation, which is an interpretive framework based on the concept of Shapley values in cooperative game theory. Shapley values are a method of assigning a degree of contribution to each parameter in a cooperative game, and SHAP builds on this foundation by applying the analysis of the contribution of features to the model output. SHAP considers the effect of each feature on different combinations of features and calculates the contribution of each feature to a certain predicted value. By weighing all possible combinations of features, the SHAP value provides a weight for each feature that explains the relative contribution of that feature to the model output.

**Figure 1:**
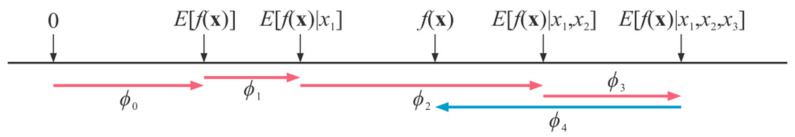
SHAP attributes.

To interpret the predictions for individual samples, Python’s SHAP package creates a visualization: The influence of each eigenvalue on the prediction result is expressed as a numerical value and summed to obtain the prediction value. As shown in Figure 1, the baseline value is the average of the predictions for the entire sample, with arrows to the left indicating a negative impact value and arrows to the right indicating a positive impact value. SHAP is the conditional expectation function of the model. *A_0_, A_1_, A_2_, A_3_,* and *A_4_* denote the weights of individual factors influencing the outcome, and E[*f*(x)], etc. denote the mathematical expectation of individual factors on the predictive model, the sum of which is *f*(x), i.e., the model prediction.

### 2.4. Implementation of ML Methods

**Figure 2:**
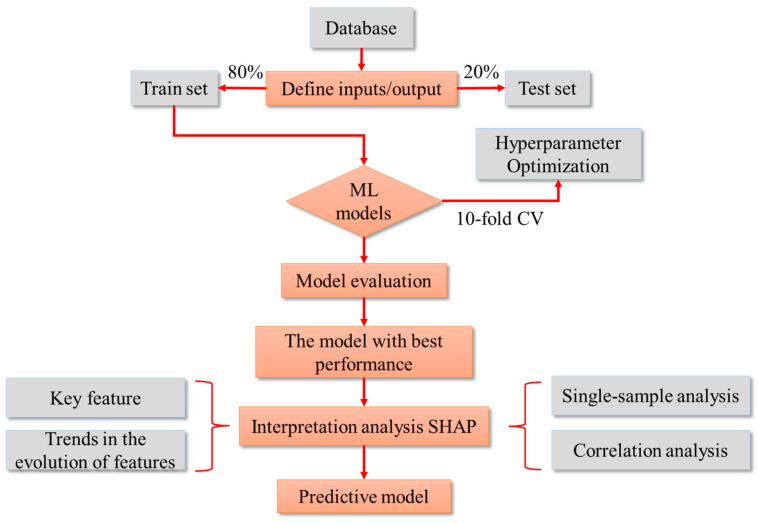
Flow chart of prediction model of concrete’s compressive strength.

As shown in Figure 2, the modeling process of this study was as follows: Firstly, the database of the samples of concrete’s compressive strength was established. Among them, the training set accounted for 80%, and the testing set accounted for 20%. Then, the parameters were adjusted to avoid model overfitting or underfitting, and the prediction model was established. Then, several prediction models were evaluated for performance and the best performing prediction model (XGBoost) was selected. Finally, the credibility of the XGBoost model was verified using (1) the key feature; (2) trends in the evolution of features; (3) a single-sample analysis; and (4) a correlation analysis.

## 3. Experimental Database

### 3.1. Selection of Input and Output Features

In this study, several parameters were selected as input features for the model; these parameters included the water–binder ratio, water, sand ratio, superplasticizer, air-entraining agent, slump, air content, and age. The model’s output was the concrete specimen’s compressive strength (v_u_).

### 3.2. Details of the Database

The database contains 228 samples. The database characteristics are as follows: (1) the specimens are 150 × 150 × 150 standard cubic blocks; (2) all of the specimens are standard cured; (3) all of the specimens are of secondary concrete, which is consistent except for the control variables, such as the selection of aggregates, and the water content of the sand, etc.

**Figure 3:**
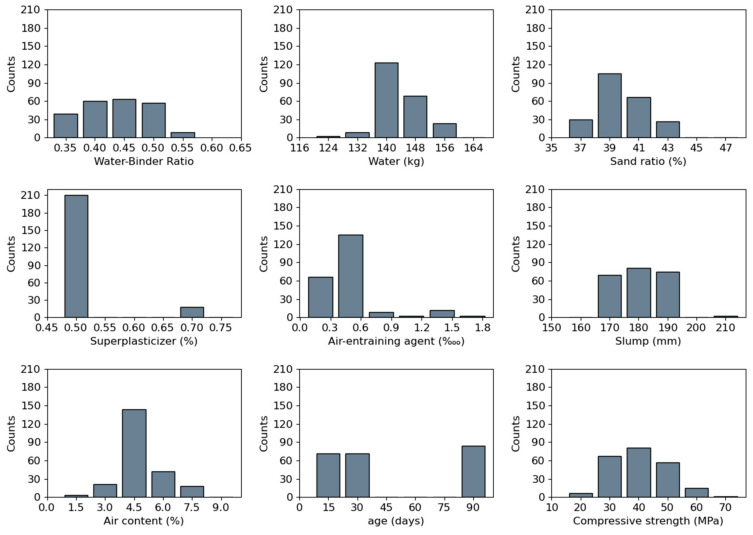
Statistical distributions of the input variables.

Table 1 describes the scope of important features of the database. The water–binder ratio ranges from 0.35 to 0.55, the water varies greatly from 124 kg/L to 154 kg/L, and the sand ratio ranges from 37% to 43%. The superplasticizer and air-entraining agent vary from 0.50% to 0.75% and from 0.20% to 1.5%, respectively. The slump and air content vary from 163 mm to 202 mm and from 1.2% to 6.4%, respectively. The ages were 7 d, 28 d, and 90 d. The experimental values of the compressive strength of concrete (*v*_test_ = *V*_test_/area) range from 17.1 MPa to 61.4 MPa. In addition, the distribution of these features can be visualized in Figure 3.

## 4. Model Results and Discussion

This section compares the performance of the models based on six algorithms, namely, the single algorithms KNN and DT, and the integrated algorithms RF, GBDT, Adaboost, and XGBoost, in predicting the compressive strength of concrete. According to Section 2.4, the important hyperparameters in the six ML models were determined, as shown in Table 2. Four evaluation metrics were selected to measure the performance of the models.

Table 3 presents the performance advantages and disadvantages of several ML models on the training and testing set. In the training set, the R^2^ of several algorithms (except KNN) is around 0.980, but XGBoost has the smallest RMSE, MAE, and MAPE values. Meanwhile, in the testing set, XGBoost has the closest R^2^ to 1 and the smallest RMSE, MAE, and MAPE values, indicating that the XGBoost model has the optimal generalization ability.

**Figure 4:**
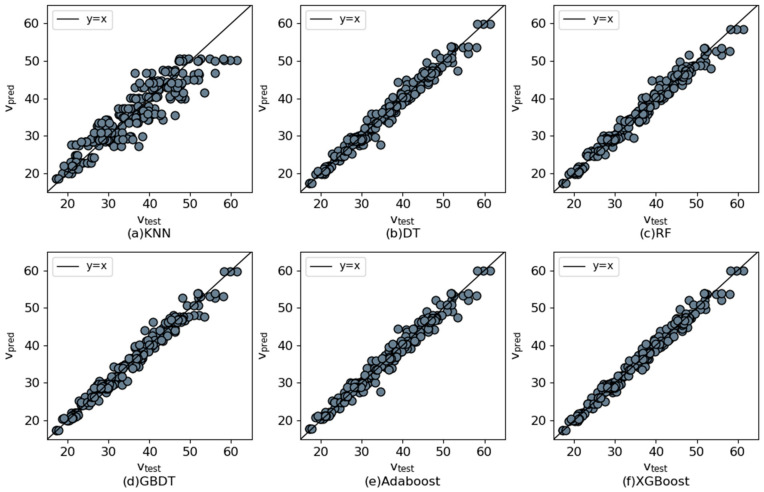
Scatter plot of compressive strength distribution.

**Figure 5:**
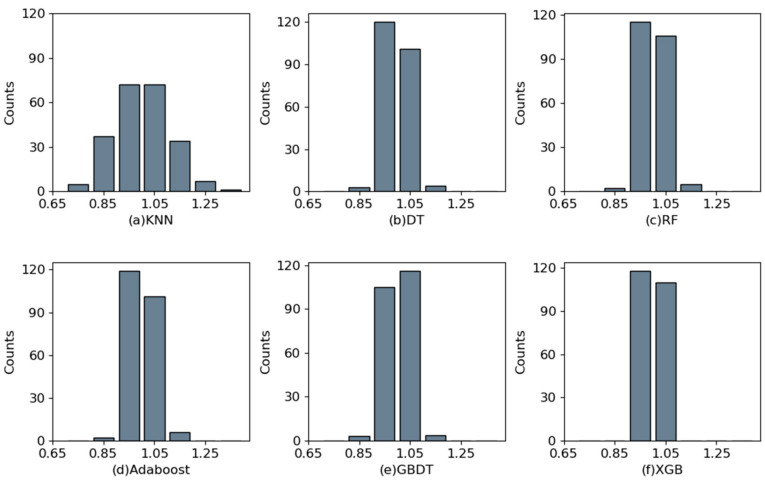
Bar graph distribution of the χ.

In Figure 4, the horizontal axis shows the experimental values, and the vertical axis shows the predicted values. The points distributed on the diagonal line indicate that the predicted values are the same as the test values. The points to the upper left of the diagonal indicate that the predicted value is greater than the experimental value. It can be seen that the scatter points of the XGBoost model are more concentrated in and around the diagonal. The KNN model has the most dispersed scatter distribution area, and several other algorithmic models have roughly the same concentration of distribution. It can be concluded that the integrated model outperforms the single ML model. The horizontal axis in Figure 5 shows the ratio of predicted to experimental values, denoted by χ; the vertical axis indicates the number of the interval. In Figure 5f, all models range from 0.95 to 1.05.

In summary, the XGBoost model has high accuracy and good generalization ability. However, the model’s prediction process is invisible, and it is unknown how the input features affect the prediction. In the next chapter, the prediction process will be uncovered through methods such as a SHAP value analysis.

## 5. Model’s Interpretable Analysis

### 5.1. Key Features

**Figure 6:**
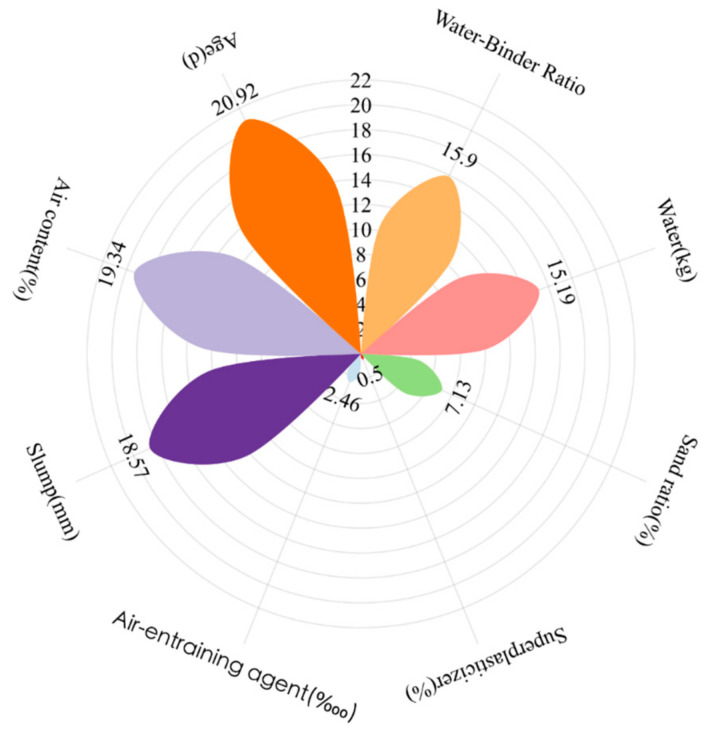
Importance of each input feature.

The absolute value of the SHAP value of each feature for all individuals is summed to give the overall feature importance. Then, the global importance of each feature is divided by the sum to obtain the relative importance value of each individual feature in the database. By ranking the importance of the features, we can know which input features are very important and which input features can be ignored. Figure 6 shows the importance of the input features.

As shown in Figure 6, the relative importance value of age (d) is 20.92%, and its effect on the concrete’s compressive strength is the largest. Then, the next most important influence on compressive strength is the air content (%), accounting for about 19.34%. The slump (mm) is the third most important input feature, and it has a value of 18.57%. The water-–binder ratio and water (kg) have the same effect on compressive strength, and their SHAP values are 15.9% and 15.19%, respectively. The sand ratio (%) also has some effect on the compressive strength, while the superplasticizer and air-entraining agent dosage have negligible impacts on the compressive strength.

### 5.2. Trends in the Evolution of Features

**Figure 7:**
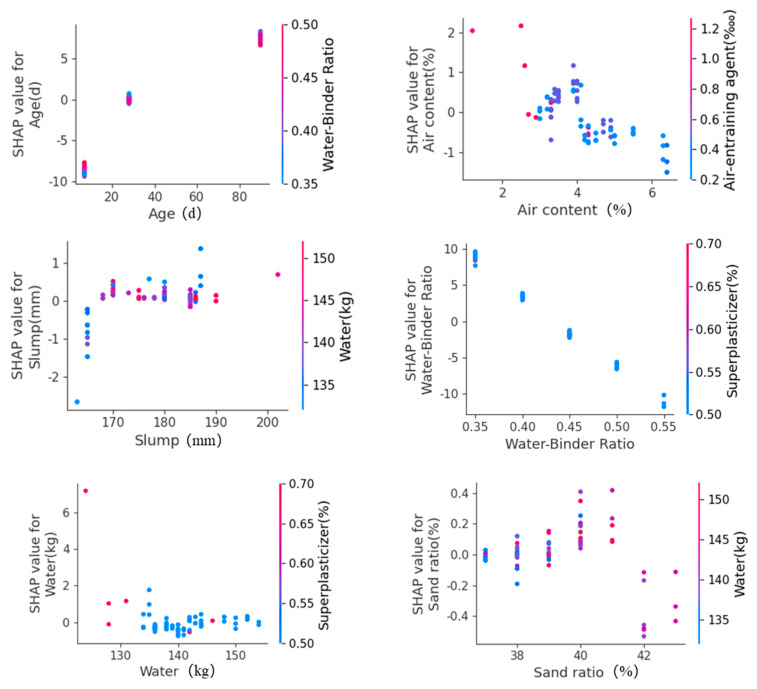
Trends in the evolution of features for important variables.

Trends in the evolution of features plots can represent the response of features to model predictions, reflecting whether the relationship between the input and output features is linear or nonlinear and monotonic or complex. Figure 7 shows the trends in SHAP values for age (d), air content (%), slump (mm), water-binder ratio, water (kg), and sand ratio (%).

It is clear that the age (d) and slump (mm) have a strong positive impact on compressive strength, while the air content (%) and water–binder ratio have a strong passive impact on compressive strength. The approximately linear relationship between the age, air content, water–binder ratio, and SHAP values shows that the higher the age, the lower the air content, and the lower the water–binder ratio, the higher the compressive strength of the concrete. The compressive strength increases faster when the slump increases from 163 mm to 170 mm, while the change in compressive strength is less pronounced when the slump is greater than 170 mm. When the water is more than 135 kg, a change in water has a negligible effect on the compressive strength. When the sand ratio is between 37% and 40%, the compressive strength increases as the sand ratio increases, while when the sand ratio is greater than 41%, the compressive strength decreases instead as the sand ratio increases; therefore, it can be concluded that a sand ratio of 40–41% is the optimum sand ratio. In conclusion, the trends in the evolution of features for important variables plot clearly reveals the impact of features on the prediction, helping us to make informed decisions using the ML model. In conclusion, the feature dependence diagram gives us an understanding of the law of the influence of input features on compressive strength, which is of great significance in guiding the actual production work.

### 5.3. Single-Sample Analysis

**Figure 8:**
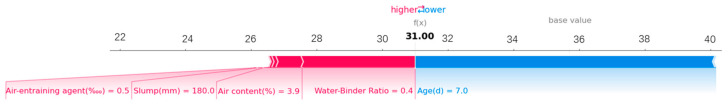
Interpretation of single-sample feature.

In this study, the test results of one of the specimens were interpreted by a single-sample model, as shown in Figure 8, which demonstrates that the SHAP value decomposes the prediction of compressive strength into the sum of the contributions of each input variable. The “base value” in Figure 8 represents the baseline value, i.e., the average of the XGBoost model predictions for all variables in the database (35.8 kN). The red arrows indicate the variables that contribute positively to the base compressive strength, while the blue arrows indicate the variables that lead to a decrease in the base compressive strength, and the lengths of the arrows indicate the incremental and decremental amounts. For this specimen, the water–binder ratio, air content, slump, and air-entraining agent content are the key variables that contribute positively to the baseline compressive strength, and their increments decrease in that order. The remaining variables, such as age, are the key variables that contribute to the reduction in the benchmark compressive strength. The algebraic summation of the increments or decrements of all the variables with the benchmark compressive strength is the model-predicted value of 31.00 kN in the figure, which is close to the test value of 31.1 kN.

### 5.4. Correlation Analysis

**Figure 9:**
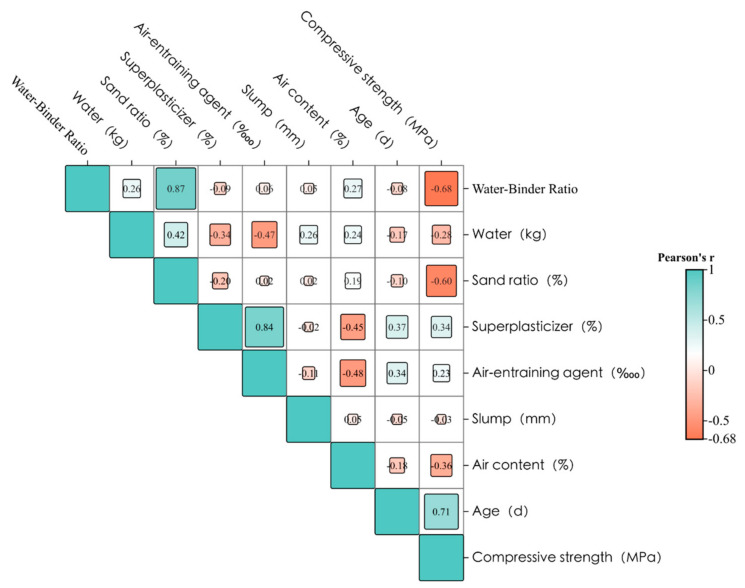
Heatmap of variable correlation.

A correlation heat map represents the correlation between the input features and the output features. The conclusions drawn from the XGBoost model were validated by correlation analysis. In Figure 9, the correlation heat map uses different value sizes and color depths to describe the correlation between different variables. A larger value indicates a higher correlation and vice versa, a lower correlation. Colors are used to distinguish between positive and negative correlations.

As shown in Figure 9, the value at the intersection of age and compressive strength is 0.71 with a light green color, indicating that the longer the age, the higher the compressive strength of the concrete. The value at the intersection of water-binder ratio and compressive strength is negative 0.68 with a darker pink color. This shows that the compressive strength decreases with the increase in the water-binder ratio. In addition, the age, water-binder ratio, air content, and slump have a significantly higher effect on the compressive strength of concrete than other variables, which is in line with Figure 6, proving that these four features are the key factors affecting the compressive strength.

## 6. Conclusions

This paper presents the application of machine learning algorithms in the prediction of concrete’s compressive strength. The theoretical background and implementation process of the algorithms are presented. A database containing 228 specimens was collected and used to train and test the ML model. The results show that the integrated algorithm outperforms the single algorithm, with XGBoost having the best performance. An interpretable analysis of the XGBoost model was also carried out to overcome the “black box” problem of previous machine learning models and increase the credibility of the model. In addition, it is possible to understand how the input parameters affect the target value, which is meaningful to guiding the actual production.

However, there were some limitations in this study: (1) the effects of aggregate and admixture types on compressive strength were not investigated; (2) this paper only analyzed the compressive strength, so important parameters such as seepage resistance, frost resistance, and other properties in the actual project were not involved; and (3) the calculation method for compressive strength was not accurate. These aspects need to be further studied.

## Figures and Tables

**Table 1:** Scope of important features of the database.

Variables	Unit	Min	Max	Mean	Median	SD
Water–Binder Ratio	-	0.35	0.55	0.44	0.45	0.06
Water	kg/L	124.00	154.00	140.30	140.00	5.78
Sand ratio	%	37.00	43.00	39.25	39.00	1.59
Superplasticizer	%	0.50	0.75	0.52	0.50	0.06
Air-entraining agent	‱	0.20	1.50	0.45	0.50	0.28
Slump	mm	163	202	177.87	180.00	8.10
Air content	%	1.20	6.40	4.08	4.00	1.00
Age	d	7.00	90.00	44.21	28.00	35.95
Compressive strength	MPa	17.10	61.40	35.69	35.85	9.58

Notes: SD = Standard deviation.

**Table 2:** Algorithm hyperparameter optimization.

Algorithm	Hyperparameter Optimization
KNN	n_neighbors = 5
DT	criterion = ‘mse’, splitter = ‘best’, min_samples_split = 2
RF	n_estimators = 68, random_state = 90, max_depth = 12
GBDT	learning_rate = 0.2, n_estimators = 5, max_depth = 3
Adaboost	max_depth = 19, learning_rate = 0.9,n_estimators = 40
XGBoost	n_estimators = 42, max_depth = 5, gamma = 0.2, learning_rate = 0.2

**Table 3:** Model performance on training and testing sets.

Models	Sets	Measures			
		R^2^	RMSE	MAE	MAPE(%)
KNN	Training	0.848	3.699	2.944	8.291
	Testing	0.725	5.079	4.334	12.133
DT	Training	0.982	1.266	0.929	2.621
	Testing	0.943	2.308	1.735	4.802
RF	Training	0.980	1.337	1.007	2.858
	Testing	0.950	2.167	1.628	4.409
GBDT	Training	0.979	1.371	1.025	2.906
	Testing	0.956	2.034	1.534	4.138
AdaBoost	Training	0.980	1.329	0.970	2.741
	Testing	0.940	2.368	1.811	5.038
XGBoost	Training	0.982	1.266	0.929	2.622
	Testing	0.966	2.307	1.734	4.801

## Data Availability

The raw data supporting the conclusions of this article will be made available by the authors on request.
